# Mechanistic Target of Rapamycin (*Mtor*) Is Essential for Murine Embryonic Heart Development and Growth

**DOI:** 10.1371/journal.pone.0054221

**Published:** 2013-01-14

**Authors:** Yi Zhu, Karla M. P. Pires, Kevin J. Whitehead, Curtis D. Olsen, Benjamin Wayment, Yi Cheng Zhang, Heiko Bugger, Olesya Ilkun, Sheldon E. Litwin, George Thomas, Sara C. Kozma, E. Dale Abel

**Affiliations:** 1 Division of Endocrinology, Metabolism, and Diabetes and Program in Molecular Medicine, School of Medicine, University of Utah, Salt Lake City, Utah, United States of America; 2 Department of Biochemistry, School of Medicine, University of Utah, Salt Lake City, Utah, United States of America; 3 Division of Cardiology, School of Medicine, University of Utah, Salt Lake City, Utah, United States of America; 4 Biomedical Center, Institute of Biology, Laboratory of Morphometry and Cardiovascular Morphology, State University of Rio de Janeiro, Rio de Janeiro, Brazil; 5 Division of Hematology-Oncology, Department of Internal Medicine, Metabolic Diseases Institute, College of Medicine, University of Cincinnati, Cincinnati, Ohio, United States of America; Instituto Gulbenkian de Ciência, Portugal

## Abstract

Mechanistic target of rapamycin (*Mtor)* is required for embryonic inner cell mass proliferation during early development. However, *Mtor* expression levels are very low in the mouse heart during embryogenesis. To determine if *Mtor* plays a role during mouse cardiac development, cardiomyocyte specific *Mtor* deletion was achieved using α myosin heavy chain (α-MHC) driven Cre recombinase. Initial mosaic expression of Cre between embryonic day (E) 10.5 and E11.5 eliminated a subset of cardiomyocytes with high Cre activity by apoptosis and reduced overall cardiac proliferative capacity. The remaining cardiomyocytes proliferated and expanded normally. However loss of 50% of cardiomyocytes defined a threshold that impairs the ability of the embryonic heart to sustain the embryo’s circulatory requirements. As a result 92% of embryos with cardiomyocyte *Mtor* deficiency died by the end of gestation. Thus *Mtor* is required for survival and proliferation of cardiomyocytes in the developing heart.

## Introduction

Mechanistic target of rapamycin (Mtor) engages in two distinct complexes (MtorC1 and MtorC2) to integrate both intracellular and extracellular signals through multiple cellular pathways to regulate cell metabolism, growth, function and survival [1]. Germline disruption of Mtor in mice leads to early embryonic lethality due to impaired proliferation and G1 arrest of embryonic stem cells [2], [3].

MtorC1 comprises Mtor, a scaffold protein raptor, the proline-rich Akt substrate of 40KD (PRAS40) and the LST8 homolog (Mlst8) [4]. MtorC1 regulates protein translation through the direct phosphorylation of S6K1 and 4E-BP1 proteins. Deletion of raptor (MtorC1) in mouse skeletal muscle results in muscle atrophy and decreased muscle function [5]. Deletion of raptor (MtorC1) in mouse adipocytes results in reduced size of the adipose depot, and mice are protected against diet-induced obesity and hypercholesterolemia as a result of increased mitochondrial uncoupling in adipocytes [6].

MtorC2 comprises Mtor, a scaffold protein rictor, hSin1, PRAS40 and Mlst8. MtorC2 was shown to act as a PDK2, which phosphorylates the Serine-Threonine kinase Akt/PKB on the Ser473 residue [7]. However, phosphorylation of Akt/PKB at Ser473 only affects Akt/PKB’s kinase activity toward a subset of downstream targets such as members of the forkhead family of transcription factors (FOXOs) [8], [9]. Tissue specific disruption of MtorC2 by rictor deletion leads to mild effects [10]. For example, mice with rictor deletion in skeletal muscle appear normal [5], and mice with rictor deletion in adipose tissue results in bigger mice due to increased whole body insulin and IGF1 levels, but the adipose tissue size was not changed [11]. Moreover MtorC2 might not be the sole PDK2: as deletion of both raptor and rictor in skeletal muscle results in elevated Akt/PKB Ser473 phosphorylation [5]. Skeletal muscle specific Mtor deletion phenocopies raptor deletion, indirectly suggesting that MtorC2 may play a minor role in skeletal muscle [12].

In the heart, MtorC1 is an important modulator of Akt/PKB regulated cardiac hypertrophy, and rapamycin treatment was able to prevent the hypertrophy induced by overexpressing a constitutively activated Akt1 [13]. However, cardiac specific overexpression of constitutively activated Mtor does not increase heart weight significantly [14]. By contrast, inducible deletion of Mtor in cardiomyocytes leads to heart failure and demise of the mouse on the basis of induction of 4E-BP1 protein, which binds to eukaryotic initiation factor 4E (eIF4E) and shuts down cap-dependent protein translation in cells [15]. The report also showed that whole body deletion of 4E-BP could double the median survival time of cardiac Mtor deficient mice from 7 weeks to 14 weeks after Mtor deletion [15].

These studies underscore the complexity with which Mtor regulates survival and function in a tissue-specific manner. Less is known about the role of *Mtor* in embryonic cardiac development. A mouse ethylnitrosourea genetic screen identified a “flat-top” phenotype of mice in which *Mtor* was mutated and kinase activity was reduced, suggesting that expansion and regionalization of the telencephalon was reliant on *Mtor* function during embryogenesis [16]. Interestingly, using *in situ* hybridization the authors observed that at embryonic day (E) 9.5, *Mtor* was widely expressed throughout the embryo, but was largely absent from the heart [16]. Although these observations suggest that *Mtor* might not be required during early heart development, *Mtor*’s role during cardiac development in mid and late gestation is unknown. This report sought to address this question by generating mice deficient for *Mtor* in cardiomyocytes at mid-gestation using α-MHC-Cre mediated *Mtor* recombination.

## Materials and Methods

### Ethics Statement

This study was carried out in strict accordance with the recommendations in the Guide for the Care and Use of Laboratory Animals of the National Institutes of Health. The protocol was approved by the Committee on the Ethics of Animal Experiments of the University of Utah (Protocol #: 09-08011). All efforts were made to minimize suffering of the mice [17].

### Mice

*Mtor^fl/fl^* mice were previously generated by Dr. George Thomas [3]. The neomycin resistance cassette in *Mtor^fl/fl^* mice was removed before they are mated to *α-MHC-Cre^tg/+^* mice. The expression pattern of Cre driven by the *α-MHC* promoter in *α-MHC-Cre^tg/+^* hearts was verified in a previous publication [18]. Because *CMtorKO* (*α-MHC-Cre^tg/+^/Mtor^fl/fl^*) mice were embryonic lethal, mice harboring heterozygous Cre and that were heterozygous for the *Mtor* loxP allele (*α-MHC-Cre^tg/+^/Mtor^fl/+^*, abbreviated as *CMtorHet*) were bred with *Mtor* loxP homozygotes to generate *CMtorKO* mice or embryos.

The primers for analyzing the loxP sites are:

Primer AC11, 5′-GCTCTTGAGGCAAATGCCACTATCACC.

Primer AC14, 5′-TCATTACCTTCTCATCAGCCAGCAGTT.

Primer AC16, 5′-TTCATTCCCTTGAAAGCCAGTCTCACC.

Doxycycline (Dox) inducible cardiac specific *Mtor* deficient mice were generated by developing compound transgenic mice harboring a reverse TetO-Cre (purchased from Jackson lab, strain number 6234), a Dox transactivator (rtTA) under the control of the α-MHC promoter [19] and two floxed *Mtor* alleles (*TetO-cre^tg/+^/α-MHC-rtTA^tg/+^/Mtor^fl/fl^*, named as *iCMtorKO*). Those *iCMtorKO* mice were administered doxycycline hyclate (Sigma, St. Louis, MO) at a dose of 4 mg/kg body weight by intraperitoneal injection at 6-week of age, and then were kept on doxycycline chow (1 g/kg) for 3 weeks to induce TetO-Cre expression, after which they were switched back to normal rodent chow for 1 more week to allow Dox washout before being sacrificed for experiments.

For timed pregnancy experiments: the day when vaginal plugs first appeared is considered 0.5 days post-coitum (E0.5). All mice used in this study were backcrossed 6 times to the C57Bl6 background. All animals described in this report were maintained and used in accordance with protocols approved by the Institutional Animal Care and Use Committee of the University of Utah.

### Western Blotting

All dissolvable proteins were extracted from whole heart lysates with a buffer containing 0.1% Triton X-100 using a tissue lyser (Qiagen, Germantown, MD). Protein concentration was determined by the Micro BCA Protein Assay kit (Pierce, Rockford, IL). Identical amounts of protein in equivalent volumes were loaded and resolved by SDS-PAGE and transferred to either PVDF (low fluorescence) or nitrocellulose membrane for immunoblot detection with specific antibodies. Detection and quantification were performed by measuring the intensity of fluorescence from secondary antibodies using the Odyssey Infrared Imaging System and accompanying software (LI-COR Biosciences, Lincoln, NE).

Primary antibody list: Tubulin and actin antibody were purchased from Sigma (St. Louis, MO); 4E-BP1 and Cre antibody were purchased from Abcam (Cambridge, MA); all other antibodies were purchased from Cell signaling (Danvers, MA).

Secondary antibody list: IRDye 800CW goat anti-Mouse was purchased from Li-Cor (Lincoln, NE). Alexa fluor goat anti-Rabbit 680 antibody was purchased from Invitrogen (Carlsbad, CA).

### Quantitative Real-time Polymerase Chain Reaction (qPCR)

qPCR was performed on cDNA reverse-transcribed from total RNA that was extracted from whole embryonic ventricles. In detail, total RNA was extracted from whole embryonic ventricles using TRIzol reagent (Invitrogen, Carlsbad, CA) using manufacturer’s protocols. RNA concentration was determined by measuring the absorbance at 260 nm using a NanoDrop 1000 spectrophotometer (NanoDrop products, Wilmington, DE). RNA quality was assessed by the ratio of absorbance measured at 260 nm and 280 nm.

0.5 µg total RNA was reverse transcribed to cDNA using the Superscript**®** III Reverse Transcriptase Kit (Invitrogen, Carlsbad, CA) using manufacturer’s protocols.

The qPCR was performed in a 384-well plate in triplicate with a standard curve, using an ABI Prism 7900HT instrument (Applied Biosystems, Foster City, CA). SYBR-green was used for fluorescence detection, and ROX was used as a reference dye. Ribosomal protein S16 gene (rpS16) was used as an internal reference. Primer sequences are listed in Table S1.

### EdU Staining, TUNEL Staining and Histology

EdU (5-ethynyl-2′-deoxyuridine) staining was used for measuring cell proliferation. EdU data is interpreted in the same way as the more commonly used BrdU (Bromo-deoxyuridine). EdU was purchased from Invitrogen (Eugene, OR). The EdU staining was based on the Click reaction [20], and has been previously validated relative to BrdU staining [21]. For embryonic studies, EdU was administrated to pregnant mice by intraperitoneal injection of 33.3 µg/g body weight, 4 hours before sacrifice. Embryos/embryonic hearts were removed immediately after sacrifice of the pregnant mouse and were fixed in 10% Zinc-formalin (Thermo Fisher Scientific, Waltham, MA) for 2 hours at room temperature, prior to paraffin embedding. Sections were cut at a thickness of 8 µM, and staining was performed according to the protocol from Invitrogen (Eugene, OR). TUNEL staining was also performed with those sections using the *In Situ* Cell Death Detection Kit, TMR Red from Roche (Indianapolis, IN) with manufacturer provided protocols. Slides were examined with an Olympus FV1000 confocal microscope. Image quantification was done by Image-Pro Plus.

Hematoxylin-eosin staining was done for analysis of cardiac wall volume and nuclei number. Masson’s trichrome staining was done for assessing fibrosis in adult hearts.

### Quantitative Stereology

The principles of stereological analysis are well documented [22] and therefore only a brief discussion of the theory is presented. According to the Cavalieri’s principle, an unbiased estimate of the volume V of an object may be obtained by slicing it from end to end, starting at a random position, with a series of *m* parallel sections a mean distance *T* apart, and measuring the area *Ai* of the object as it appears on the *ith* section, i.e: V[Cardiac walls] = ∑T(A1+A2+…+Am).

The number of myocyte nuclei and of interstitial nuclei (including fibroblasts, the nuclei of pericytes, macrophages and round cells, and also endothelial cells of capillary vessels) per volume (D_i_) was determined using optical dissectors on 10 µM-thick sections. The numerical density of myocytes in the *i*th section: D_i_ = Q^−/^(T*A_i_), where Q^−^ is the number of cells on the chosen section (n_i_) of the tissue sample minus the remaining number of cells on the next section of the sample (n_i+1_): Q^−^ = n_i_-n_i+1_. The total nuclei number is N = D*V[cardiac walls], D is the mean of D_i_.

### X-gal Staining

For the cre activity reporter assay, whole-mount X-gal staining was done at 37°C for 2 hours. After being fixed in 2% paraformaldehyde for 1 hour on ice, the whole embryos/embryonic hearts were washed and incubated with X-gal staining solution containing 5 mmol/L K_4_Fe(CN)_6_ (Potassium Ferrocyanide), 5 mmol/L K_3_Fe(CN)_6_ (Potassium Ferricyanide), 2 mmol/L MgCl_2_, 0.01% NP-40, 0.01% deoxycholate and 0.1% X-gal in PBS. Then the stained embryos were embedded in paraffin and sectioned at 8 µM thickness. The sections were counter-stained with nuclear fast red (Sigma, St. Louis, MO) for visualization.

### Electron Microscopy (EM)

Samples were initially fixed in 2.5% glutaraldehyde and 1% paraformaldehyde, and then post-fixed in 2% osmium solution. After fixation, samples were stained with electron-opaque uranyl acetate aqueous solution and dehydrated through a graded series of ethanol washes. Next, stained samples were embedded and cut for transmission electron microscopy.

### Adult Mouse Echocardiography and Fetal Ultrasound

Mice were anesthetized with isoflurane and placed on a heated stage (37°C). Chest hair was then removed with a topical depilatory agent before the echocardiogram. Short and long axis two-dimensional guided M-mode images were taken with a 13 MHz linear probe from GE Medical Systems (Milwaukee, WI) for the measurements of left ventricular dimensions and wall thickness. Fractional shortening [%] is calculated as 100*[(LVDd - LVDs)/LVDd]. LVDd: left ventricular dimension-diastolic; LVDs: left ventricular dimension-systolic.

Similarly, for fetal ultrasound, pregnant mice were anesthetized with isoflurane and placed on a heated stage. The echocardiograms of embryos were recorded with a 40-MHz transducer of the 660 ultrasound machine from VisualSonics (Toronto, Ontario, Canada) [23]. The genotype correlation with ultrasound findings was done by matching the position of the embryos in the uterus relative to the position in the abdomen, as determined by the ultrasound.

### Statistical Analysis

All values are shown as mean ± standard error. Data sets with two groups were compared with Student’s t-test unless specified. Data sets with three or more groups were initially compared with one-way ANOVA. If a statistical significance existed, Bonferroni’s test was then used as a post-hoc test. A P≤0.05 was considered significant. Statistics were performed with Microsoft EXCEL, Origin 8 or GraphPad Prism. Plots were drawn using Origin 8 or GraphPad Prism. Figures were created using Adobe Illustrator CS5.

## Results

### Deletion of *Mtor* by Alpha-MHC-Cre Results in Embryonic Lethality

**Figure 1:**
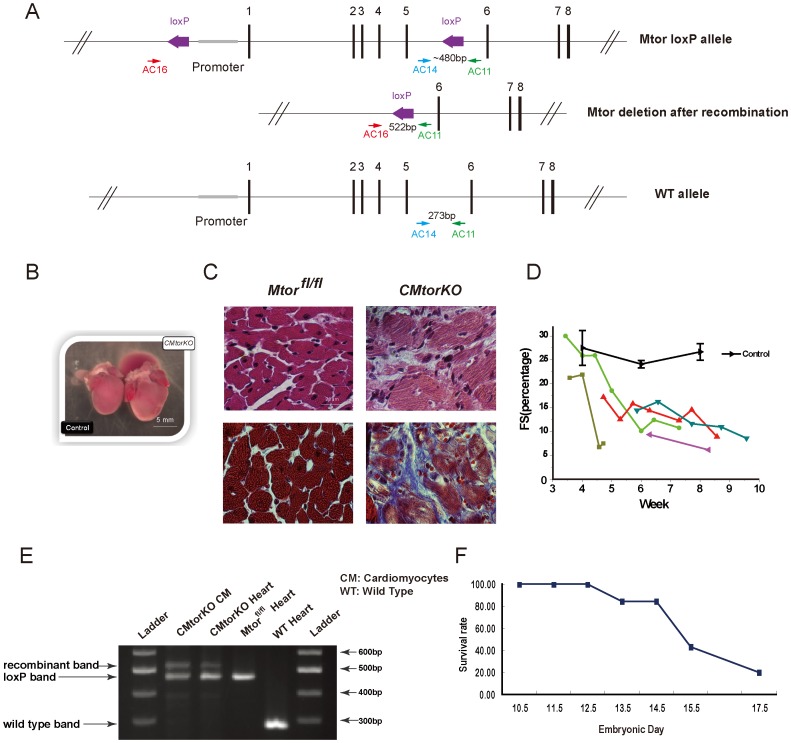
Deletion of *Mtor* by alpha-MHC-Cre is embryonic lethal. (A) Schematic showing conditional *Mtor* allele and location of the loxP sites. The position of AC11, AC14 and AC16 primers and the size of the DNA segments amplified by AC primer pairs are illustrated. (B) Representative picture of an 8-week old (surviving) *CMtorKO* heart and an *Mtor^fl/fl^* control. (C) Histological analysis of 8-week old *Mtor^fl/fl^* and *CMtorKO* hearts: upper panel is H&E staining; lower panel is Masson’s trichrome staining. (D) Fractional shortening (FS) measured by echocardiography, black line represents an average of 5 *Mtor^fl/fl^* control mice, and each colored line represents a single *CMtorKO* mouse. (E) Agarose gel electrophoresis of AC11, AC14 and AC16 PCR products using DNA isolated from cardiomyocytes (CM) obtained from a *CMtorKO* heart, *CMtorKO* heart tissue, *Mtor^fl/fl^* heart tissue and wild type (WT) heart tissue respectively. (F) Survival curve of *CMtorKO* embryos.

The *Mtor* allele was modified by inserting LoxP sites proximal to the *Mtor* promoter and downstream of exon 5 [3]. Thus Cre-mediated recombination will delete the *Mtor* promoter and the first 5 exons. Primers AC16 and AC11 recognize DNA sequences that are 5′ and 3′ respectively of each LoxP (Fig. 1A). Thus PCR amplification of genomic DNA using AC11 and AC16 primers would generate a 522 bp DNA fragment following Cre-mediated recombination. Primer pair AC11 and AC14 will amplify either the non-recombined floxed allele (∼ 480 bp) or the wildtype allele (273 bp).

Cardiomyocyte-restricted *Mtor* deficient mice (*CMtorKO*, genotype α*-MHC-Cre^tg/+^/Mtor^fl/fl^*) were generated by crossing mice harboring a heterozygous alpha-myosin heavy chain (α-MHC) Cre transgene that were also heterozygous for the *Mtor* loxP allele (*α-MHC-Cre^tg/+^/Mtor^fl/+^*) with homozygous *Mtor* loxP (*Mtor^fl/fl^*). Assuming Mendelian inheritance, 25% of mice born should be *CMtorKO* mice. However, only a small number of *CMtorKO* mice (12 of 590 weaned mice) were alive at the time of weaning, suggesting that 92% of the *CMtorKO* mice died either in utero or in the perinatal period. No deaths of *CMtorKO* mice were noted in the perinatal period suggesting that most of the mortality occurred in utero. Surviving *CMtorKO* mice exhibited dramatic cardiac hypertrophy (Fig. 1B) and intense replacement fibrosis and myocyte disarray (Fig. 1C). Their left ventricular (LV) ejection fraction progressively declined overtime until their demise (Fig. 1D). All surviving *CMtorKO* mice died by 10-week of age from cardiac failure.

DNA was isolated from heart tissue or isolated cardiomyocytes of surviving 5–6 week old *CMtorKO* mice with heart failure. Cardiac DNA was subjected to PCR amplification with primers AC11, AC14 and AC16. *CMtorKO* mouse hearts revealed a 522 bp band confirming the presence of the recombined allele. The presence of a lower band that was amplified by primers AC11 and AC14 indicates persistence of the unrecombined allele in whole heart as well as in isolated cardiomyocytes. Whereas the existence of this unrecombined allele in whole heart DNA could reflect LoxP alleles in non-cardiac cells such as fibroblasts, the persistence of the band in isolated cardiomyocytes although potentially consistent with contamination, also suggests low-level or incomplete recombination in surviving cardiomyocytes (Fig. 1E).

In light of these preliminary observations of perinatal or embryonic lethality and evidence of partial *Mtor* allelic recombination in cardiomyocytes isolated from the small number of surviving mice, timed pregnancy experiments were performed and embryos harvested at varying times post-coitus. All *CMtorKO* embryos were alive at embryonic day 12.5 (E12.5) and began dying around E13.5. By E15.5 and E17.5, only 43% (26 out of 60) and 20% (1 out of 5) of *CMtorKO* embryos respectively, were still alive (Fig. 1F).

### Expression of *Cre* Recombinase in the Embryonic Heart Resulted in *Mtor* Deletion, Decreased Proliferation and Increased Apoptosis between E10.5 and E12.5, and as a Result Reduced Heart Size by E12.5

**Figure 2:**
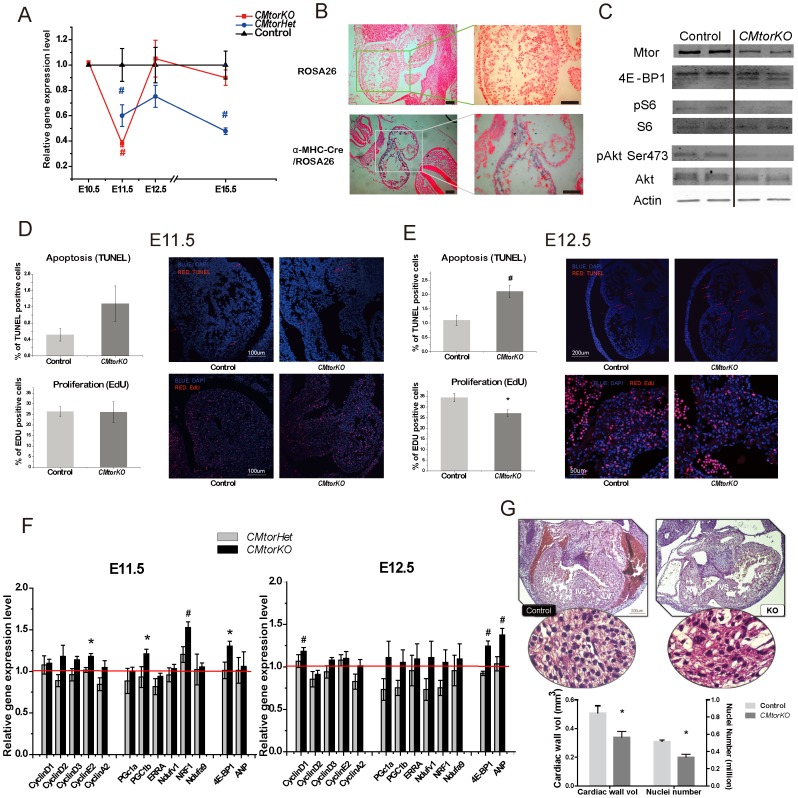
Deletion of *Mtor* between E10.5 and E12.5 reduced heart size by E12.5. (A). *Mtor* mRNA from *CMtorKO* hearts and *CMtorHet* hearts at various embryonic stages (n = 6–8). (B) Representative pictures (left: low magnification, right: high magnification) of X-gal staining of ROSA26 (upper panel) or α-MHC-Cre/Rosa26 (lower panel) embryonic heart at E10.5, counterstained with nuclear fast red. Scale bar = 100 µM. (C) Western blots of Mtor and Mtor downstream signaling molecules in E12.5 embryonic hearts. The three 4E-BP bands from the top to the bottom are hyper-phosphorylated, phosphorylated and non-phosphorylated. (D) Representative TUNEL staining (n = 7) and Edu Staining (n = 3–6) of E11.5 embryonic hearts (right), quantification is shown on the left. Arrows indicate TUNEL positive nuclei. (E) Representative TUNEL staining (n = 4–5) and Edu staining (n = 3–4) of E12.5 embryonic hearts (right), quantification is shown on the left. Arrows indicate TUNEL positive nuclei. (F) Relative gene expression levels of E11.5 (left) and E12.5 (right) *CMtorHet* (Het) and *CMtorKO* (KO) embryonic hearts, the gene expression levels of their littermate controls are set to 1 (n = 6–8). (G) Quantification of cardiac wall volume and cardiac nuclei number of E12.5 embryonic control and *CMtorKO* (KO) hearts (n = 3–4). *: p≤0.05 vs. control, #: p≤0.01 vs. control.

Prior studies have shown that α-MHC-Cre is turned on at ∼ E9.5 [24]. Consistent with the onset of α-MHC-Cre expression and subsequent *Mtor* recombination, we observed a peak reduction of *Mtor* mRNA in E11.5 *CMtorKO* embryonic hearts. At this age, there was a 62.1% reduction of *Mtor* mRNA in *CMtorKO* hearts compared to their littermate *Mtor^fl/fl^* controls (Fig. 2A). The partial reduction of *Mtor* mRNA is hypothesized to result from mosaic expression of α-MHC-Cre, as previously demonstrated [18], and confirmed by X-gal staining of the hearts of α-MHC Cre mice crossed with ROSA26 mice (Fig. 2B). However, the decline in *Mtor* mRNA was transient and *Mtor* mRNA levels reverted to control levels at E12.5 and were maintained through E15.5 in surviving *CMtorKO* hearts (Fig. 2A). These findings suggest that a cohort of cardiomyocytes in which *Mtor* was not deleted may proliferate to reconstitute the cardiomyocyte population. In contrast, in *Mtor* heterozygous embryonic hearts, *Mtor* mRNA expression levels first dropped by approximately 40.0% compared to littermate controls at E11.5, and remained around 50%–70% of their littermate control levels through E15.5 (Fig. 2A). Consistent with these mRNA data, Mtor protein and phosphorylation of MtorC1 downstream signaling molecules 4E-BP1 and S6 were reduced in E12.5 *CMtorKO* hearts relative to control hearts, and Akt Ser473 phosphorylation was also reduced, suggesting impaired MtorC2 signaling in *CMtorKO* hearts (Fig. 2C).

We next measured cardiomyocyte proliferation and rates of apoptosis at various stages of embryonic development. At E10.5, prior to the decline of *Mtor* mRNA, cardiomyocyte proliferation rates as measured by Edu staining [21] did not change in *CMtorKO*, nor was any difference observed in apoptosis levels as measured by TUNEL staining (data not shown). At E11.5, the proliferation of *CMtorKO* embryonic cardiomyocytes remained the same as their littermate controls and apoptosis was not statistically different (Fig. 2D). At E12.5, there was a decrease in proliferation and an increase in apoptosis (Fig. 2E). The delay in the onset of the proliferation defect and increase in apoptosis relative to the reduction in *Mtor* mRNA likely reflects the time needed for degradation and turnover of already expressed Mtor proteins in E11.5 *CMtorKO* hearts.

At E11.5, despite a decrease in *Mtor* mRNA, expression levels of various cyclins (*cyclin D1, cyclin D2, cyclin D3, cyclin E2, cyclin A2*) in *CMtorKO* hearts were not changed. These cyclin genes also remained unchanged at E12.5, suggesting that the defect in proliferation in *CMtorKO* hearts is not regulated via transcriptional repression of cyclins (Fig. 2F). Mtor was previously shown to regulate mitochondrial biogenesis by transcriptional mechanisms in skeletal muscle [25]. However mitochondrial related genes (*PGC1α, PGC1β, ERRα, Ndufv1, Ndufa9*) were not changed in E11.5 and E12.5 *CMtorKO* hearts (Fig. 2F). Mitochondrial morphology was also unchanged in E11.5 and E12.5 *CMtorKO* hearts relative to controls (Fig. S1). *4E-BP1* mRNA was induced in E11.5 and E12.5 *CMtorKO* hearts by 30% and 24% respectively, but was not changed in *CMtorHet* hearts (Fig. 2F). Relative to controls, *ANP* mRNA levels were unchanged in *CMtorKO* hearts at E11.5, but were significantly increased by 38% compared to control hearts at E12.5, indicating a hemodynamic stress response in *CMtorKO* hearts at E12.5. *CMtorHet* hearts had normal expression of *ANP* at both E11.5 and E12.5 (Fig. 2F).

To quantify nuclei number in embryonic hearts, we prepared paraffin embedded sections of the whole embryonic heart, and stained sections with hematoxylin and eosin (H&E). Nuclei number and cardiac wall volume were estimated using well-established stereological analysis methods for the heart [22]. At E12.5, there was a 33% reduction of cardiac wall volume and a 34% reduction of total cardiac nuclei number compared to controls (Fig. 2G); calculated cardiomyocyte volume (size) was not changed (Fig. S2). In E12.5 embryonic hearts, fibroblasts only contribute up to 8.3% of the total cell population [26]. Thus the decrease in nuclei number in E12.5 embryonic hearts is likely the result of a reduction in cardiomyocyte number. Moreover, as the α myosin heavy chain promoter is specifically expressed in cardiomyocytes, other cell types should not be affected. Total heart RNA content was also reduced by 35% (p<0.01) in *CMtorKO* hearts at E12.5, consistent with the decline in cardiac nuclei number and cardiac wall volume.

### Restored Proliferation and Normal Apoptosis Rate in E14.5 CMtorKO Hearts

**Figure 3:**
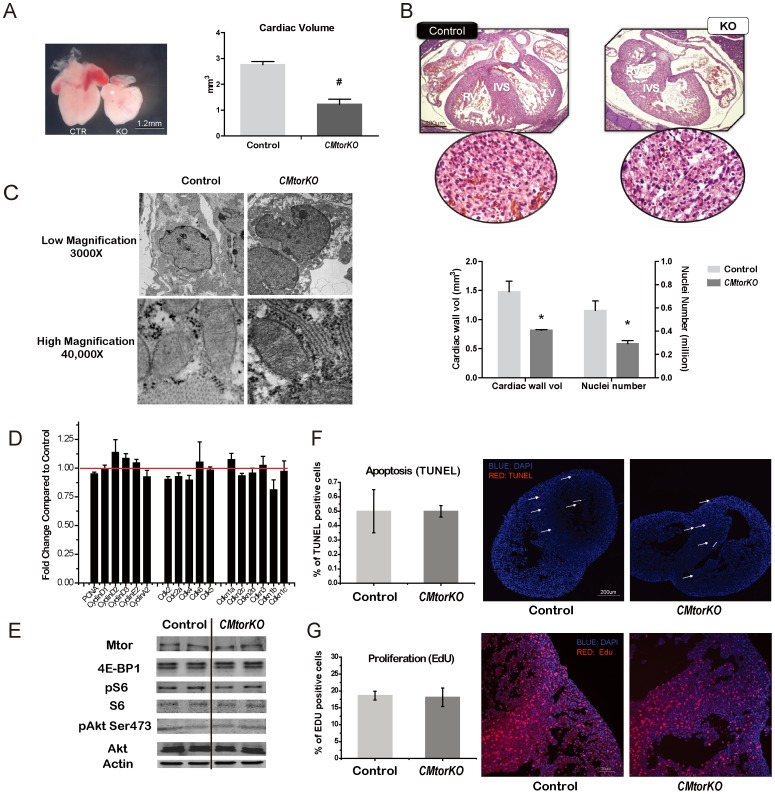
Restored proliferation and normal apoptosis rate in E14.5 *CMtorKO* hearts. (A) A representative picture of E14.5 *Mtor^fl/fl^* (CTR) and *CMtorKO* (KO) embryonic hearts (left), and calculated cardiac volume (right) (n = 3–4). (B) Quantification of cardiac wall volume and cardiac nuclei number of E15.5 control and *CMtorKO* (KO) embryonic hearts from live embryos (n = 3–4). (C) Representative EM pictures of control and *CMtorKO* embryonic heart from live embryo at E15.5. (D) Relative gene expression levels of E15.5 *CMtorKO* embryonic hearts from live embryos compared to their littermate *Mtor^fl/fl^* controls (n = 8). (E) Western blots of Mtor and Mtor downstream signaling molecules in E14.5 embryonic hearts. (F) Representative TUNEL staining for E14.5 embryonic hearts (right) (n = 3–4), quantification is shown on the left. Arrows indicate TUNEL positive nuclei. (G). Representative Edu staining for E14.5 embryonic hearts (right) (n = 3), quantification is shown on the left. *: p≤0.05 vs. control, #: p≤0.01 vs. control.

*CMtorKO* embryos did not die immediately following *Mtor* deletion. Instead at E14.5, 85% of the *CMtorKO* embryos were still alive. Morphometric analyses of E14.5 *CMtorKO* hearts revealed a 50% reduction in volume, using the simplified calculation of Volume (V) = 4/3πr^3^ with measured radius (r) (Fig. 3A).

By E15.5, only 43% of *CMtorKO* embryos were still alive. Cardiac wall volume and cardiac nuclei number were reduced by 45% and 49% respectively compared to the controls, suggesting ongoing loss of cardiomyocytes between E12.5 to E15.5, while the gross morphology of H&E stained *CMtorKO* hearts was normal at E15.5 (Fig. 3B).

Although more than 50% of *CMtorKO* embryos died by E15.5, surviving *CMtorKO* embryos displayed normal mitochondrial crista morphology and normal nuclei structure when their cardiomyocytes were examined by electron microscopy (EM) (Fig. 3C). Furthermore, expression of genes encoding various cyclins, cyclin dependent kinases, and cyclin dependent kinase inhibitors were not changed in E15.5 *CMtorKO* hearts that were isolated from viable embryos. Expression of mitochondrial OXOPHOS genes or autophagy related genes were also not changed (Fig. 3D). Consistent with maintained Mtor mRNA levels (Fig. 2A), Mtor proteins and phosphorylation of MtorC1 downstream signaling molecules 4E-BP1 and S6 were not changed in E14.5 *CMtorKO* hearts relative to control hearts, and Akt Ser473 phosphorylation was also not reduced, suggesting intact MtorC2 signaling in E14.5 *CMtorKO* hearts (Fig. 3E). In addition, at E14.5, TUNEL staining showed no increase of apoptosis in *CMtorKO* hearts (Fig. 3F) and cardiomyocyte proliferation rates were also normal (Fig. 3G).

### Development of Cardiac Dysfunction and the Death of CMtorKO Embryos

Surviving embryonic hearts showed normal valves and no obvious developmental defect in any of the four cardiac chambers, indicating *Mtor* deletion does not impair cardiac development per se.

**Figure 4:**
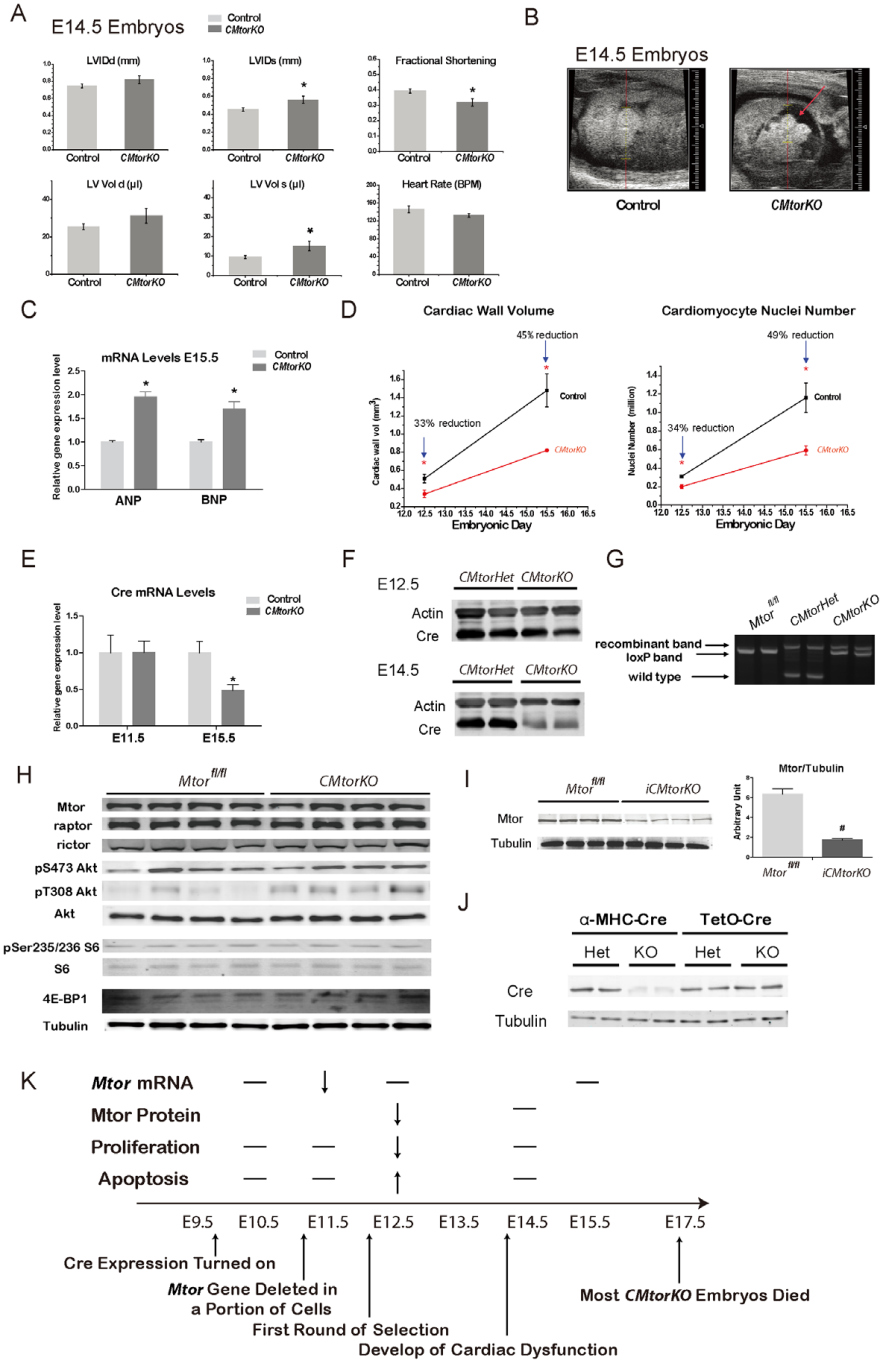
Development of cardiac dysfunction and the death of *CMtorKO* embryos. (A) Fetal echocardiography measurements of E14.5 embryonic hearts (n = 9–17). LVIDd: left ventricular interior dimension-diastole; LVIDs: left ventricular interior dimension-systole; LV vol d: left ventricular volume-diastole; LV vol s: left ventricular volume-systole. (B) Representative cardiac echocardiogram of a control embryonic heart (left) and a *CMtorKO* embryonic heart (right). The arrow indicates pericardial fluid in the *CMtorKO* embryo. (C) *ANP* and *BNP* mRNA levels in E15.5 *CMtorKO* hearts from live embryos (n = 8). (D) A summary of cardiac wall volume and cardiac nuclei number from E12.5 to E15.5. (E). *Cre* recombinase transcripts levels in *CMtorHet* and *CMtorKO* hearts at E11.5 and E15.5 (n = 8). (F). Western blots of Cre recombinase in E12.5 and E14.5 embryonic hearts. (G). Agarose gel electrophoresis of AC11, AC14 and AC16 PCR products using DNA isolated from E14.5 *Mtor^fl/fl^* hearts, *CMtorHet* hearts and *CMtorKO* hearts. (H). Western blots of Mtor, raptor, rictor and Mtor downstream signaling molecules in 6–9 week old (adult) failing *CMtorKO* hearts. (I). Western blot of Mtor protein from adult, doxycycline-induced Mtor deficient hearts (*iCMtorKO*) (left) and densitometric quantification (right) (n = 4–6). (J). Western blot of Cre recombinase protein from 8-week old *CMtorHet*, *CMtorKO* hearts (α-MHC-Cre) and 10-week old *iCMtorHet*, *iCMtorKO* hearts (TetO-Cre). (K). A summary of cellular and physiological events in *CMtorKO* embryos and suggested model of how artificial selection by expressing α-MHC-Cre in mouse heart leads to embryonic lethality. “^__^“ indicates no change, blank means not measured at the time point. *: p≤0.05 vs. control, #: p≤0.01 vs. control.

Thus we decided to ask a fundamental question whether the death of the embryos was caused by cardiac failure. Cardiac function of embryos was measured by fetal ultrasound/echocardiography at E14.5, one day before massive deaths of embryos. The echocardiography was performed in a blinded fashion after which embryos were sacrificed and genotyped. Embryos that were analyzed in this way had the following genotypes: *Mtor^fl/+^, Mtor^fl/fl^, α-MHC-Cre^tg/+^/Mtor^fl/+^* (*CMtorHet*) and *α-MHC-Cre^tg/+^/Mtor^fl/fl^* (*CMtorKO*). Cardiac function (ejection fraction, fractional shortening) and cardiac dimensions (LVIDd, LVIDs) were the same for *Mtor^fl/+^* and *Mtor^fl/fl^* embryos. Also western blotting showed that *Mtor^fl/+^* and *Mtor^fl/fl^* adult hearts had similar levels of Mtor proteins (data not shown), suggesting no hypomorphic effect of the *Mtor* floxed allele. Therefore, we pooled data from these two genotypes and used them as controls. Cardiac function of *CMtorHets* was normal compared to control embryos (Table 1). In contrast, *CMtorKO* hearts had about 20% reduction of cardiac function measured by fractional shortening. *CMtorKO* hearts were also dilated, as suggested by increased systolic left ventricular internal dimension (LVIDs) and systolic LV volume (Fig. 4A and Table 1). Some *CMtorKO* embryonic hearts showed pericardial fluid accumulation suggesting a terminal condition associated with inadequate cardiac output that failed to support continued embryonic development (Fig. 4B, Movie S1, S2). Expression of *ANP* and *BNP* was increased by 95% and 70% respectively in E15.5 surviving *CMtorKO* embryonic hearts, which is consistent with findings of cardiac dysfunction as suggested by fetal echocardiography (Fig. 4C). Increased ANP and BNP mRNA was not associated with changes in the cardiac chamber maturation genes in E15.5 hearts (Fig. S3). These findings suggest that ANP induction is a consequence of altered cardiac function versus altered left ventricular maturation.

**Table 1:** Fetal echocardiographic evaluation of embryonic cardiac function at E14.5.

Parameter	Control	CMtorHet	CMtorKO
No. of embryos	17	9	12
IVSd (mM)	0.247±0.011	0.198±0.008#	0.200±0.011#
LVIDd (mM)	0.746±0.024	0.739±0.047	0.819±0.047%
LVPWd (mM)	0.221±0.015	0.199±0.008$	0.175±0.011*
LVIDs (mM)	0.452±0.018	0.466±0.048$	0.561±0.043*
RVd (mM)	0.779±0.060	0.769±0.043&	0.892±0.040%
RVs (mM)	0.474±0.044	0.493±0.039$	0.581±0.041*
Fractional Shortening (%)	39.46±1.39	37.60±3.16$	31.92±2.43*
Ejection Fraction	0.730±0.017	0.703±0.040$	0.630±0.034*
LV Vol d (µl)	25.48±1.63	25.35±3.38	31.29±3.82%
LV Vol s (µl)	9.45±0.76	10.59±2.51	15.05±2.41*
SV (µl)	16.03±1.06	14.77±1.37	16.24±1.95
HR (BPM)	145.65±8.02	145.10±13.61	132.24±4.25
Cardiac Output	2.300±0.184	2.160±0.294	2.192±0.305

Abbreviations: IVSd: interventricular septum thickness-diastole; LVIDd: left ventricular interior dimension-diastole; LVPWd: left-ventricular posterior wall thickness at diastole; LVIDs: left ventricular interior dimension-systole; RVd: right ventricular dimension-diastole; RVs: right ventricular dimension-systole. Data shown are mean ± SEM.

# p<0.01 VS Control,

* p<0.05 VS Control,

% p<0.10 VS Control,

& p<0.05 VS CMtorKO,

$ p<0.10 VS CMtorKO.

Cre recombinase mRNA and protein levels were reduced between E14.5–E15.5 in *CMtorKO* hearts relative to *CMtorHet* hearts (Fig. 4E, F). PCR amplification of genomic DNA from E14.5 *CMtorHet* hearts with AC primers revealed equal amounts of the recombinant and wildtype alleles with no evidence of the floxed allele, suggesting robust recombination of the floxed *Mtor* allele in *CMtorHet* hearts. In contrast, the same primer pair amplified the unrecombined floxed allele to an equivalent extent as the recombined allele (Fig. 4G), suggesting partial recombination because of reduced Cre expression or reduced number of cells that express Cre recombinase. We posit that this might account for preservation of *Mtor* mRNA expression at E15.5 (Fig. 2A).

It therefore appears that the failure of the heart is not a direct consequence of ongoing cell death or a persistent defect in cardiomyocyte proliferation. Instead, the progressive loss of cardiomyocytes and reduced cardiac size prior to E14.5 (Fig. 4D) that failed to sustain the circulatory requirements of the growing embryo caused cardiac dysfunction and failure.

## Discussion

We believe that the cardiac phenotypes in *CMtorKO* embryos derive from the mosaic expression of Cre recombinase [18] (Fig. 2B), which leads to a wave of cell death by apoptosis and impaired proliferation at E12.5. The transient nature of this event is supported by the increase in *Mtor* mRNA in *CMtorKO* hearts to levels seen in littermate controls between E11.5 and E12.5 (Fig. 2A). We posit that, around E10.5 or earlier, a subset of cardiomyocytes expressed Cre recombinase leading to recombination of the floxed *Mtor* alleles, thereby resulting in reduced *Mtor* mRNA at E11.5. As previously synthesized Mtor protein became degraded in these cells, apoptosis and a reduction in proliferation ensued. This first wave of *Cre* expression is the strongest, and then the expression of the α-MHC promoter declines [27], allowing cardiomyocytes in which *Mtor* was not deleted to proliferate to replace dead cells with *Mtor* deletion, accounting for the increase in cardiac wall volume and cardiomyocyte number (albeit reduced relative to controls) from E12.5 to E15.5 in *CMtorKO* hearts (Fig. 4D). The mechanism by which *Mtor* deficient cells exhibited a growth disadvantage is likely due to a reduction in phosphorylation of S6 and 4E-BP1, both of which regulate protein synthesis in cells. The increase in *4E-BP1* mRNA expression in *CMtorKO* embryonic hearts contributes to accumulation of non-phosphorylated 4E-BP1, which serves as a brake on protein translation. Similar accumulation of non-phosphorylated 4E-BP1 was also observed in adult hearts with inducible *Mtor* deletion, and was partially responsible for development of cardiac contractile dysfunction in those mice [15]. Eventually, before the demise of the *CMtorKO* embryos, most of the cells remaining in *CMtorKO* hearts had normal levels of *Mtor* mRNA which accounts for the normal proliferation rate and lack of any increase in apoptosis in cardiomyocytes from E14.5 *CMtorKO* hearts. However *CMtorKO* hearts remain smaller compared to their littermate controls, which cannot sustain the circulatory requirements of the embryo (Fig. 4 A–D and K).

This model is also supported by total cardiac levels of *Cre* mRNA at two different time points. At E15.5, *CMtorKO* hearts exhibited approximately a 50% reduction of *Cre* mRNA compared to their littermate heterozygous *Mtor* deficient siblings, although *Cre* mRNA levels were similar at E11.5 (Fig. 4E). Similarly, Cre protein levels were maintained at E12.5, but were reduced at E14.5 (Fig. 4F). These data suggest that a loss of *Cre* expressing cardiomyocytes occurred after E12.5 leading to selection of cardiomyocytes with presumably lower levels of Cre recombinase, which then results in a reduction in recombination efficiency of *Mtor* loxP alleles in *CMtorKO* embryos (Fig. 4G). Given the fact that *CMtorKO* hearts have two *Mtor* floxed alleles and both can be theoretically recombined, the persistence of a *Mtor* loxP band with a similar intensity to the recombinant band is consistent with less than a 50% recombination rate in the *CMtorKO* hearts at E14.5 (Fig. 4G). In fact, mice with germline heterozygous deficiency of *Mtor* have no overt phenotype [3]. Moreover, mice with cardiomyocyte restricted heterozygous specific *Mtor* deletion have normal heart weights, and are fertile (Fig. S4), suggesting that a single *Mtor* allele is sufficient to maintain cardiac structure and function. Thus if lower *Cre* expression results in a loss of only one floxed allele in a population of cardiomyocytes, those cardiomyocytes with heterozygous *Mtor* deletion should proliferate normally. This may also explain the persistent albeit reduced Cre expression occurring concurrently with normal levels of *Mtor* between E14.5–E15.5 in *CMtorKO* hearts.

Further support for this model comes from the observation that those *CMtorKO* mice that survived after weaning did not show a reduction in Mtor protein and Mtor downstream signaling (Fig. 4H). Those mice did show an increase in Akt T308 phosphorylation in their hearts, which could be secondary to heart failure. In contrast, when the same floxed *Mtor* allele was exposed to Cre recombinase in adult hearts using a doxycycline inducible TetO-Cre transgene, Mtor protein content was reduced in whole heart homogenates by more than 70% (Fig. 4I). Western blotting revealed a reduction of Cre protein in *CMtorKO* hearts relative to either *CMtorHet* or *iCMtorKO* hearts (Fig. 4J), further supporting the hypothesis of selection of low *Cre* expressing cells specifically in *CMtorKO* hearts.

The *α-MHC-Cre* transgene has been widely used and delivers near complete cardiac specific deletion of many floxed alleles such as the insulin receptor (IR) [28]. We believe that the mosaicism of *Cre* expression is more likely the result of asynchronous initial expression of *Cre* at the time cardiac progenitors begin to express α myosin heavy chain proteins. If *α-MHC-Cre* is used to delete a gene that is not dispensable for cell autonomous survival, then cumulative deletion will be seen, leading to a robust protein reduction in adult hearts.

In conclusion, we have shown that *Mtor* is essential for cardiac development and growth during embryogenesis despite a low expression of *Mtor* in the heart compared to other organs [16]. Our data suggest that mosaic expression of Cre recombinase led to loss of a subset of cardiomyocytes via mechanisms that involve increased cell death and decreased proliferation. This placed selection advantage on cells that express lower levels or no Cre, which were initially sufficient to sustain some degree of cardiac growth and development. However, this critical early loss of cardiomyocytes placed *Mtor* deficient hearts in jeopardy so that as the embryo grows the reduced cardiac mass is not sufficient to support its circulatory requirements.

## Supporting Information

Figure S1 **Mitochondrial morphology revealed by electron microscopy (EM) in E11.5 and E12.5 control and** ***CMtorKO*** **hearts.**
(PDF)

Figure S2 **Average cardiomyocyte volume calculated from cardiac wall volume and nuclei numbers in E12.5 and E15.5 control and** ***CMtorKO*** **hearts.** n = 3–4.
(PDF)

Figure S3 **Expression of cardiac chamber maturation genes in E15.5** ***CMtorKO*** **hearts.** A.U. = arbitrary unit, and control group is set at 1. n = 8.
(PDF)

Figure S4 **Body weight and heart weight of** ***CMtorHet*** **mice were not changed compared to wild type mice or** ***Mtor^fl/fl^*** **mice (n = 7–8), 6-week of age, females.**
(PDF)

Table S1 **Primer sequences used for qPCR.** All primers are shown in this order: Sequence of forward (Fwd) primer (5′→3′) Sequence of reverse (Rev) primer (5′→3′) GenBank reference sequences and Primer-BLAST were used to design the primers. To avoid unspecific amplifications, most primer sequences span at least one intron and were blasted against the mouse genome. Dissociation curves were used for all primer pairs to ensure single product amplification.
(XLS)

Movie S1 **Fetal ultrasound of a control heart at E14.5.** A normal contracting heart is observed.
(AVI)

Movie S2 **Fetal ultrasound of a** ***CMtorKO*** **heart at E14.5.** Pericardial fluid is observed.
(AVI)
