# Supplementary figures and images for: Mechanistic Target of Rapamycin (Mtor) Is Essential for Murine Embryonic Heart Development and Growth
Source: PLoS One. 2013 Jan 14;8(1):e54221. doi: 10.1371/journal.pone.0054221 (PMC3544830; doi:10.1371/journal.pone.0054221)

50,000 X

Control

*CMtorKO*

E11.5

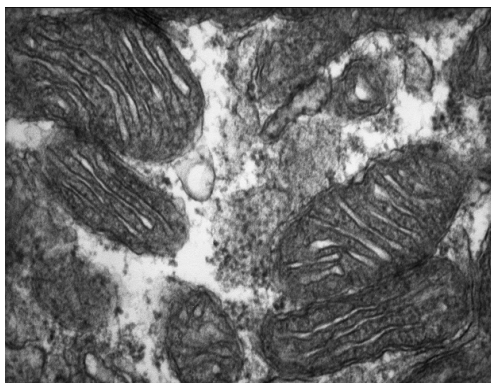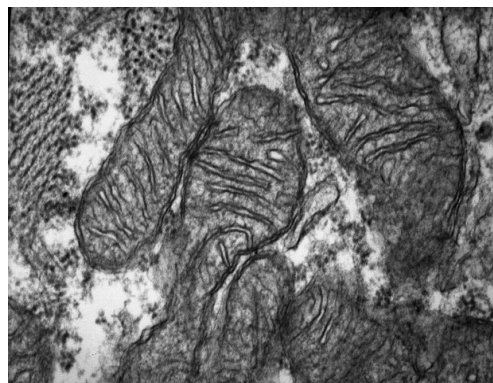

E12.5

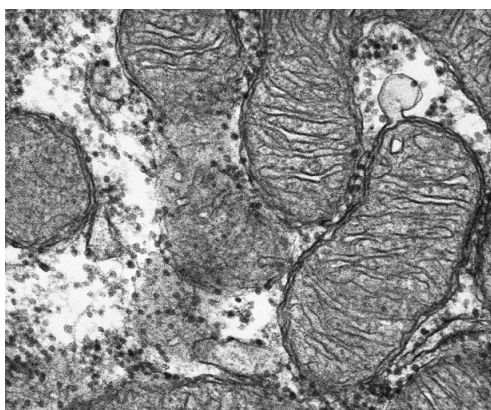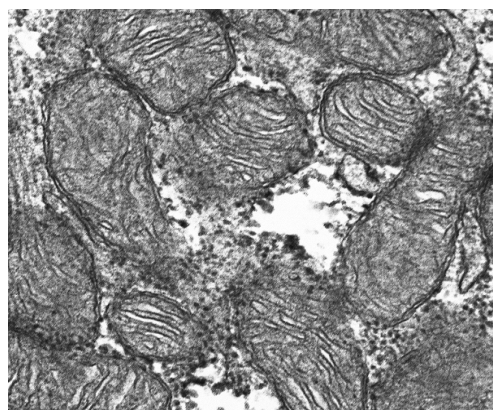

Supplement: Figure S1 — Mitochondrial morphology revealed by electron microscopy (EM) in E11.5 and E12.5 control and CMtorKO hearts. (PDF) [file pone.0054221.s001.pdf]

# Cardiomyocyte Volume

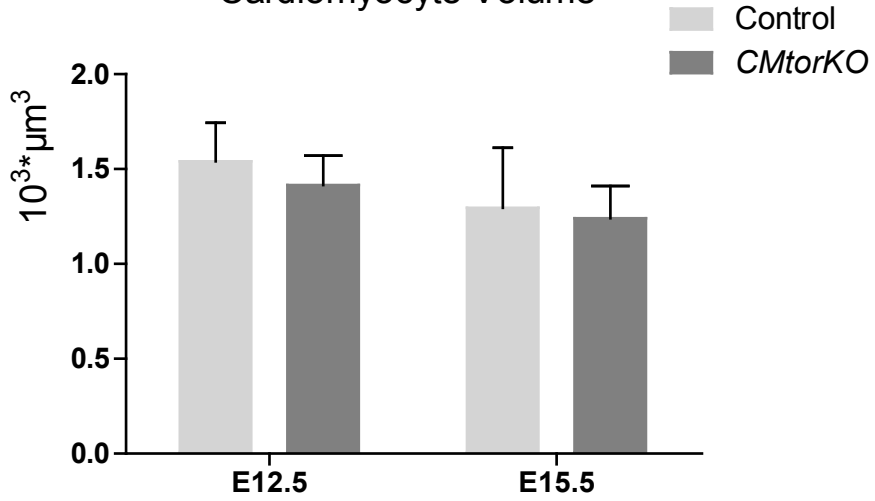

Supplement: Figure S2 — Average cardiomyocyte volume calculated from cardiac wall volume and nuclei numbers in E12.5 and E15.5 control and CMtorKO hearts. n = 3–4. (PDF) [file pone.0054221.s002.pdf]

**Rxra mRNA**

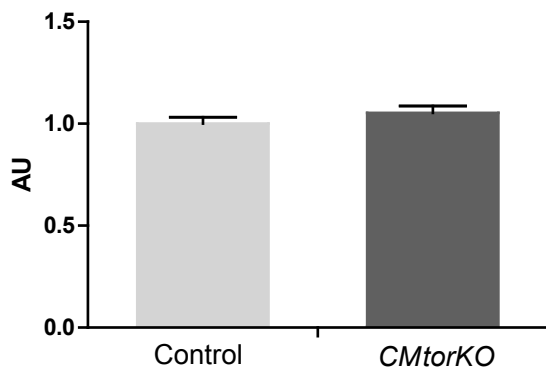

**Mycn mRNA**

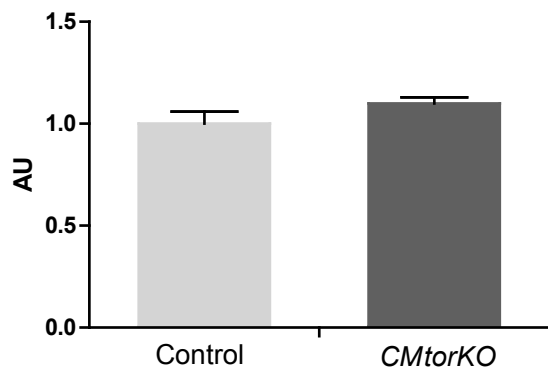

**Tef-1 mRNA**

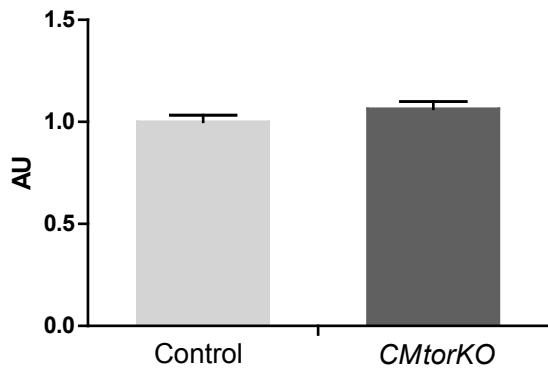

**Tbx5 mRNA**

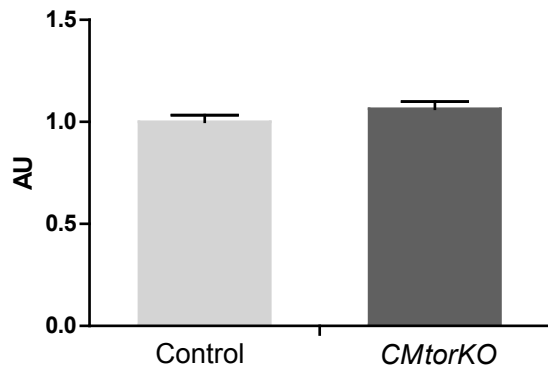

Supplement: Figure S3 — Expression of cardiac chamber maturation genes in E15.5 CMtorKO hearts. A.U. = arbitrary unit, and control group is set at 1. n = 8. (PDF) [file pone.0054221.s003.pdf]

Body Weight

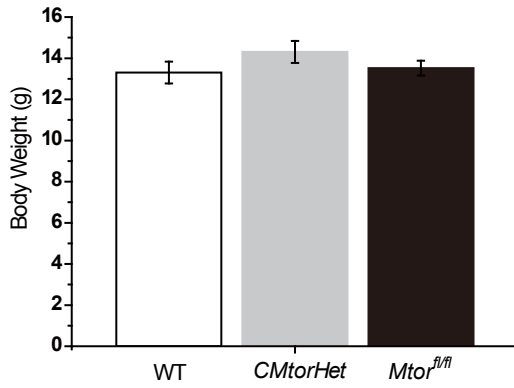

Heart Weight/Body Weight

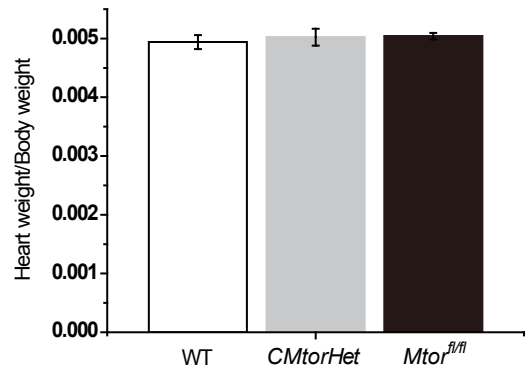

Supplement: Figure S4 — Body weight and heart weight of CMtorHet mice were not changed compared to wild type mice or Mtorfl/fl mice (n = 7–8), 6-week of age, females. (PDF) [file pone.0054221.s004.pdf]
